# Ecotoxicological Effects of Ibuprofen on Plant Growth of *Vigna unguiculata* L.

**DOI:** 10.3390/plants9111473

**Published:** 2020-11-02

**Authors:** Leonard Wijaya, Mohammed Alyemeni, Parvaiz Ahmad, Ahmed Alfarhan, Damia Barcelo, Mohamed A. El-Sheikh, Yolanda Pico

**Affiliations:** 1 Department of Botany and Microbiology, College of Science, King Saud University, P.O. Box 2455, Riyadh 11451, Saudi Arabia; mnyemeni@ksu.edu.sa (M.A.); pahmad@ksu.edu.sa (P.A.); alfarhan@ksu.edu.sa (A.A.); dbcqam@cid.csic.es (D.B.); melsheikh@ksu.edu.sa (M.A.E.-S.); 2 Water and Soil Quality Research Group, Department of Environmental Chemistry, IDAEA-CSIC, Jordi Girona 18-26, 08034 Barcelona, Spain; 3 Environmental and Food Safety Research Group (SAMA-UV), Desertification Research Centre CIDE (CSIC-UV-GV), Faculty of Pharmacy, University of Valencia, Av. Vicent Andrés Estellés s/n, 46100 Burjassot, Valencia, Spain; Yolanda.Pico@uv.es

**Keywords:** ibuprofen, *Vigna unguiculata*, pharmaceutical pollutant, seed germination, plant growth, enzyme activities, IBU toxicity

## Abstract

Despite the prevalence of the common pharmaceutical ibuprofen (IBU) in water and sediments worldwide, the effects of IBU on plants are largely unknown. This study was designed to assess the ecotoxicological effects of emerging pharmaceutical pollutant IBU on plant growth and development in a series of toxicity experiments using cowpea (*Vigna unguiculata*). Plant growth parameters (morphological and physicochemical) were investigated under a series of IBU concentrations (0, 400, 800, 1200, 1600, 2000 ppm IBU). IBU exposure reduced the shoot and root lengths, fresh and dry weights, leaf area, and chlorophyll a and b, carotenoid, total chlorophyll, mineral (K and Mg), glutathione reductase, and soluble protein contents. Simultaneously, increases in Ca and Mn contents, sodium translocation from roots to shoots, H_2_O_2_, malondialdehyde, superoxide dismutase, catalase, ascorbate peroxidase, and IBU uptake were observed. The amount of bioaccumulated IBU varied between 7% and 8%. IBU was translocated from roots to shoots with a translocation factor of 3–16%. The IC_50_ values for biomass and plant length were 1253 and 1955 ppm IBU, respectively, which is much higher than the reported levels of IBU in the environment. This study demonstrates that cowpea plants develop several morphological and physicochemical adaptations to cope under ibuprofen stress; environmentally relevant concentrations of IBU are unlikely to produce negative impacts.

## 1. Introduction

Pharmaceutical drug research focuses on how drugs affect target organs as well as ensuring the drug is persistent, retaining its chemical structure to perform the desired therapeutic effect [1]. However, the possible physiological and ecological effects of these compounds on nontarget species and ecosystems are largely unknown [2,3]. Therefore, the release of pharmaceutical compounds into the environment is of great concern for both environmental and human health [4]. Many pharmaceuticals used in human medical care are not completely reduced by the human body; more than 50% of the intake is excreted in feces or urine, entering the sewage system unchanged or slightly transformed [5]. Furthermore, many unused and expired drugs are directly discharged to the environment without any treatment due to a lack of standard protocols or regulatory policies for the disposal of pharmaceuticals, especially in the Middle East [6].

Ibuprofen is a nonsteroidal anti-inflammatory drug (NSAID) that is used to treat pain, common colds, fever, inflammation, migraines, and reduce human prostate cancer cell proliferation. Globally, approximately 44,347 metric tons of ibuprofen (IBU) was used in 2019 [7]. IBU represents one of the most widespread pharmaceuticals found in surface waters and sediments around the world. A growing human population and increasing health care trend have elevated the amount of consumed and expired IBU, leading to a steady increase of IBU in wastewaters. The compound may negatively affect humans, animals, microbes, and plants [8,9,10,11,12,13]. Although wastewater treatment plants can remove up to 80% of the IBU in water, a significant amount remains in the effluent, which can negatively impact the receiving environment [14]. Additionally, the repeated application of sewage sludge and use of reclaimed water for irrigation can also lead to the accumulation of IBU in agricultural areas. In Al-Hassa, Saudi Arabia, researchers found accumulated IBU at average concentrations of 0.5 ppb in wastewater, 2.4 ppb in sediment, 6 ppb in soil, and 13.52 ppb in plant samples [15,16]. Despite reports of IBU accumulation in the ecosystem, the ecotoxicological effects of IBU on plants have not been well studied.

Cowpea (*Vigna unguiculata* (L.) Walp.) is one of the most important food legumes in semi-arid regions of the tropics [17]. Over 14 million ha of agricultural land worldwide are occupied by cowpea, with a yearly production of 4.4 Mt [18]. The fast growth, sensitivity, and developmental characteristics of cowpea make this crop a suitable organism for assessing the toxicological effects of pollutants. This study evaluates the effects of IBU on the vegetative stage of cowpea using a series of toxicity experiments.

## 2. Results and Discussion

**Figure 1:**
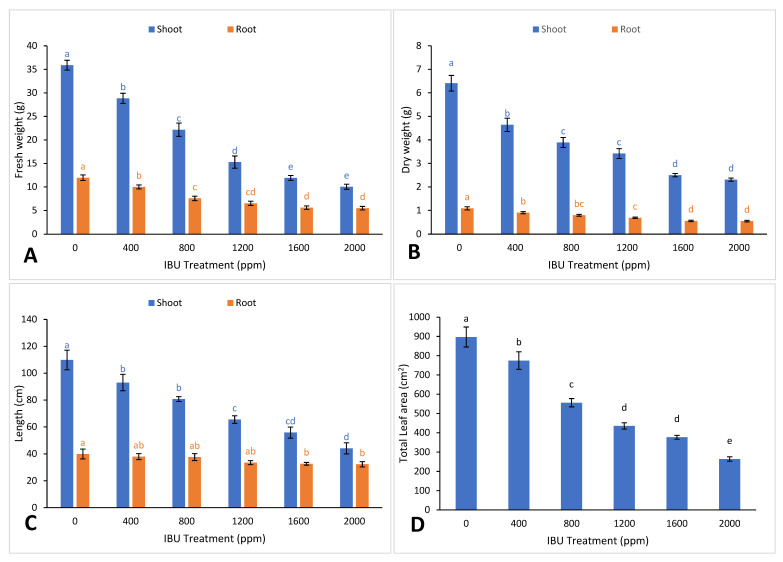
Effect of ibuprofen (IBU) treatments on (**A**) fresh weight, (**B**) dry weight, (**C**) length, and (**D**) leaf area of *Vigna unguiculata* on day 50. Different superscripts above the bars indicate significant differences at each measured time point (*p* < 0.05). All values are mean ± SE of three replicates.

Plants suffer from abiotic stress due to pollution and environment-related factors. Plants respond to stress in complex and marvelous ways, including morphological and physiological changes [19]. In *V. unguiculata* plants, growth and biomass reduction were the main outcomes of exposure to high concentrations of IBU (Figure 1A–D). The IC_50_ value for biomass (IBU 1253 ppm, *R*^2^ 0.948, *p*-value 4.01 × 10^−17^) and plant length (IBU 1955 ppm, *R*^2^ 0.974, *p*-value 6.20 × 10^−22^) were calculated using regression. The shoot and root length, biomass weight, and total leaf area of IBU-treated plants decreased dose-dependently as a result of the IBU cytotoxic effect [20], chlorophyll breakdown [10], nutrient imbalance [21], and inhibition of cell division [11]. Other researchers have also reported severe reductions in the morphological parameters of *Salix alba* [22], *Typha latifolia, Juncus effuses* [23], and *Phragmites australis* [24] exposed to IBU stress.

**Figure 2:**
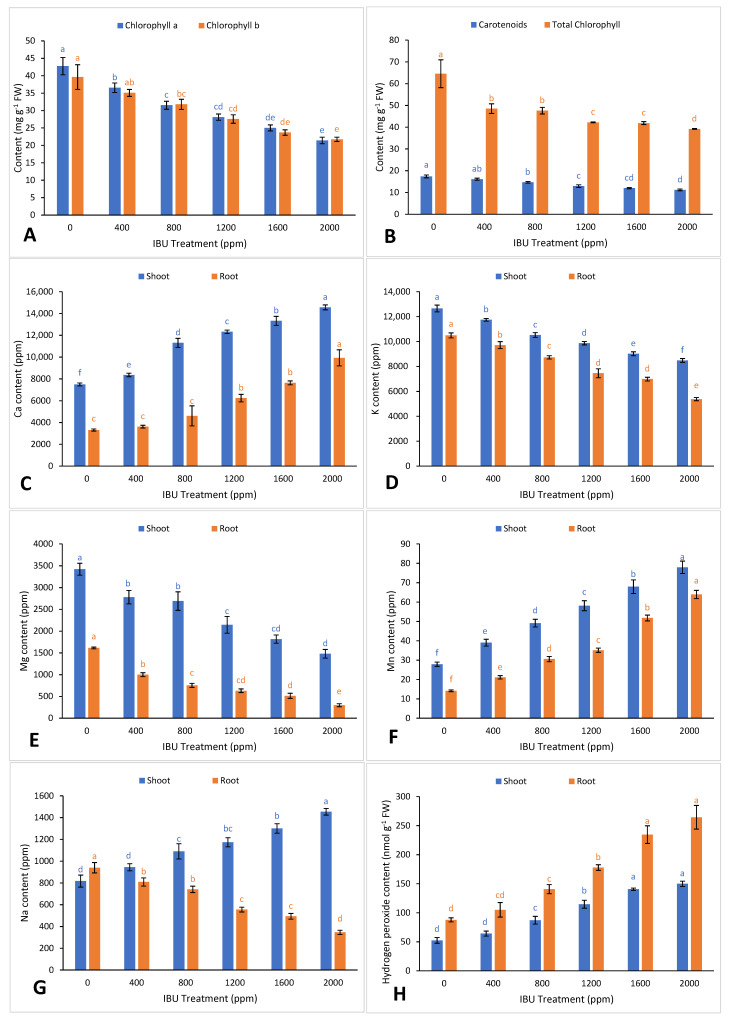
Effect of ibuprofen (IBU) treatments on (**A**) chl a and b, (**B**) car and total chl, (**C**) calcium, (**D**) potassium, (**E**) magnesium, (**F**) manganese, (**G**) sodium, and (**H**) H_2_O_2_ in *V. unguiculata* on day 50. Different superscripts above the bars indicate significant differences at each measured time point (*p* < 0.05). All values are mean ± SE of three replicates.

In the current study, IBU application induced a significant decline in chlorophyll a, chl b, total chlorophyll, and total carotenoid contents in *V. unguiculata* leaves (Figure 2A,B). The observed reduction in photosynthetic pigments may be attributed to several mechanisms, namely, the inhibition of biosynthesis or breakdown of pigments or their precursor molecules [24], reduction in photosynthetic activity in leaf quantum yield ΦPSII [22], breakdown of the thylakoid and chloroplast envelope due to its lipophilic properties [24], and decreased leaf area [25,26]. The alteration of chlorophyll and carotenoid contents by IBU was previously reported by Moro et al. [10] in microalgae *Scenedesmus rubescens*, Iori et al. [22] in *Salix alba*, Kotyza et al. [24] in *Phragmites australis*, and Wrede [27] in *Ceratophyllum demersum*.

The application of IBU to *V. unguiculata* plants caused a nutrient imbalance (Figure 2C–G). Significant increases in calcium content were observed in the shoots and roots of the plants. Calcium plays a structural role within cells and signals plant responses such as osmotic regulation [28,29]. Disturbance in Ca balance (above 10,000 ppm) changes the photochemical phase and affects photosystem II (PSII), which reduces the stability and aggregation of chlorophyll molecules in the antenna complex [30], regulation of electron transport, and synthesis of NADP and ATP [31]. IBU also lowered the potassium content in the shoots and roots of *V. unguiculata*, which affects stomata regulation, transpiration, and osmoregulation [32]. IBU exposure reduced the magnesium content in the shoots and roots of *V. unguiculata*. Low magnesium inhibits nitrogen metabolism and reduces the photosynthesis rate due to slow light reaction in the stroma [33,34], which causes reductions in biomass and photosynthesis and increases biochemical disorders [21]. In this study, IBU was shown to increase the accumulation of manganese in the shoots and roots of *V. unguiculata*. Excess Mn is toxic to the plant, which manifests as a reduction in biomass and photosynthesis and biochemical disorders such as oxidative stress [21]. Long-term treatment with IBU increased sodium translocation from the roots to shoots of *V. unguiculata*. Sodium accumulation in the shoots leads to early aging of adult leaves, reduction in the photosynthetic mechanism, and toxicity symptoms, which cease protein synthesis and interfere with enzyme regulation [35].

**Figure 3:**
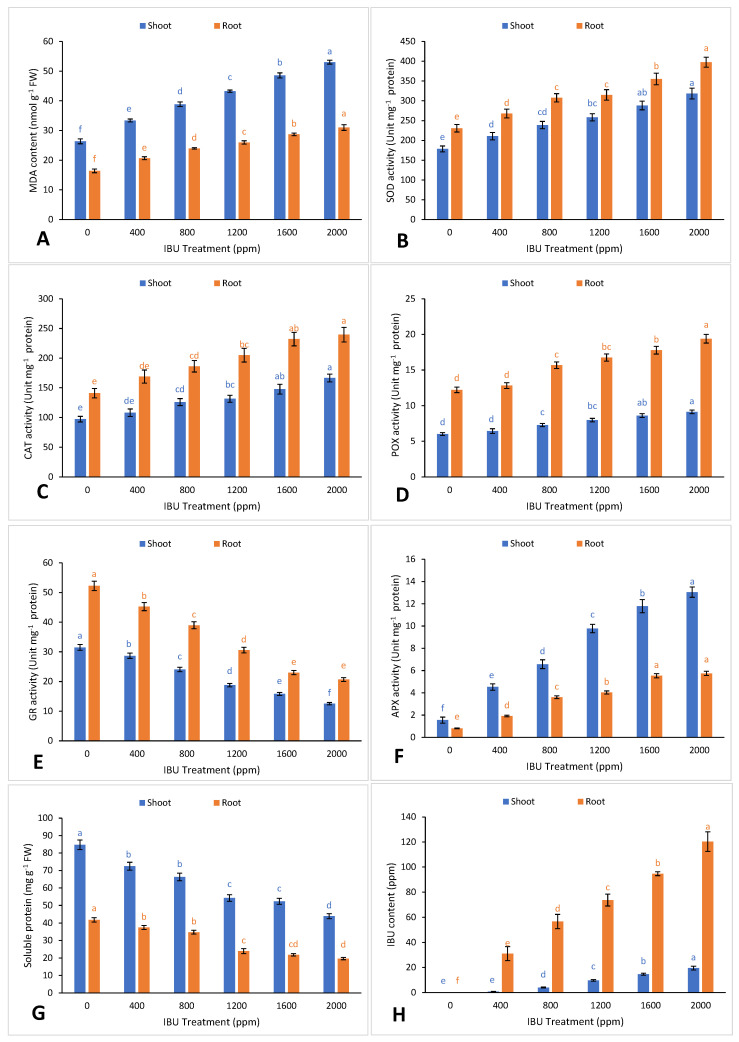
Effect of ibuprofen (IBU) treatments on (**A**) malondialdehyde (MDA), (**B**) superoxide dismutase (SOD), (**C**) catalase (CAT), (**D**) peroxidase (POX), (**E**) glutathione reductase (GR), (**F**) Soluble protein, (**G**) ascorbate peroxidase (APX), and (**H**) IBU content in *V. unguiculata* on day 50. Different superscripts above the bars indicate significant differences at each measured time point (*p* < 0.05). All values are mean ± SE of three replicates.

Pharmaceutical stress triggers an oxidative burst in subcellular compartments, especially in the mitochondria and chloroplasts, through the accumulation of reactive oxygen species (ROS). These ROS compounds have the ability to disrupt cell integrity by damaging lipids, proteins, and DNA, ultimately leading to cell death [36]. In the present study, IBU application increased hydrogen peroxide ROS content in *V. unguiculata* shoots and roots (Figure 2H). Excess ROS in plants results in lipid peroxidation of the cell membrane, which produces malondialdehyde (MDA), a reactive mutagen and a marker of oxidative stress [37]. Increased MDA content in the shoots and roots of *V. unguiculata* plants was observed after IBU exposure (Figure 3A), indicating that the properties of IBU can cause oxidative damage. Our findings support previous reports by Almohisen [38] and Kummerová et al. [39], in which pharmaceutical applications resulted in oxidative stress, increasing H_2_O_2_ and MDA content in *Vigna radiata* and *Lemna minor*, respectively.

**Figure 4:**
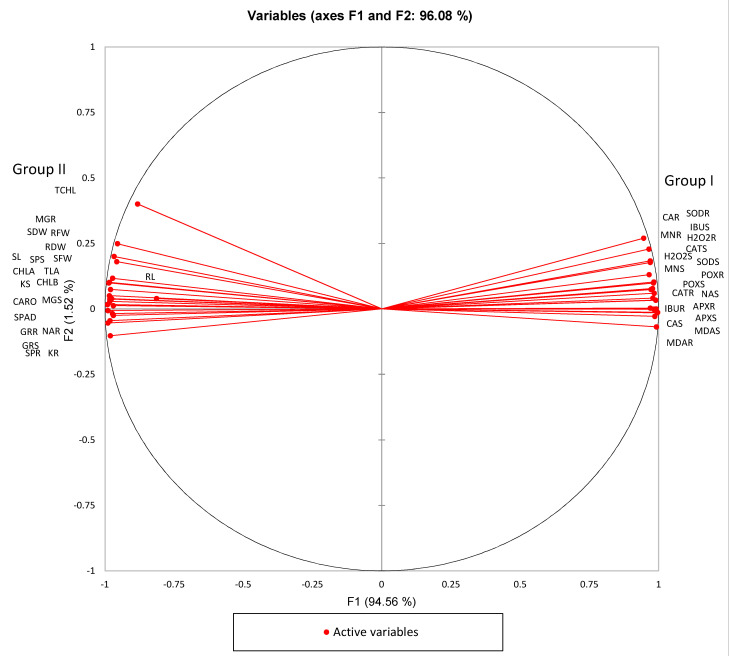
PCA (principal component analysis) of IBU effects on *V. unguiculata*. SL, shoot length; RL, root length; SFW, shoot fresh weight; SDW, shoot dry weight; RFW, root fresh weight; RDW, root dry weight; TLA, total leaf area; SPAD, leaf SPAD; CHLA, chlorophyll a; CHLB, chlorophyll b; CARO, total carotenoids; TCHL, total chlorophyll; CAS, shoot calcium; CAR, root calcium; KS, shoot potassium; KR, root potassium; MGS, shoot magnesium; MGR, root magnesium; MNS, shoot manganese; MNR, root manganese; NAS, shoot sodium; NAR, root sodium; H2O2S, shoot H_2_O_2_; H2O2R, root H_2_O_2_; MDAS, shoot MDA; MDAR, root MDA; SODS, shoot SOD; SODR, root SOD; CATS, shoot CAT; CATR, root CAT; POXS, shoot POX; POXR, root POX; GRS, shoot GR; GRR, root GR; SPS, shoot soluble protein; SPR, root soluble protein; APXS, shoot APX; APXR, root APX; IBUS, shoot ibuprofen; IBUR, root ibuprofen.

Excess ROS are scavenged by the enzymatic and nonenzymatic components of the plant-antioxidant defense system [40]. Some of the antioxidative enzymes that are directly involved in ROS detoxification are catalase (CAT), superoxide dismutase (SOD), ascorbate peroxidase (APX), glutathione peroxidase (GPX), and glutathione reductase (GR) [40]. Our results show that ibuprofen uptake leads to substantial increases in antioxidant activities, which contribute to plant stress tolerance. The activities of antioxidative enzymes SOD, CAT, APX, and GR were enhanced with exposure to IBU (Figure 3B–F). SOD accelerates the conversion of superoxide anion (O_2_^−^) into molecular oxygen (O_2_) and H_2_O_2_ [41]. H_2_O_2_ is catalyzed by GPX, GR, CAT, and APX to convert it into non-toxic components such as oxygen, H_2_O, and other alcohols [42]. Elevated antioxidant levels due to pharmaceutical stress were also documented in *V. radiata* [38], *Typha ssp.* [43], *Populus alba* [44], and *Lemna minor* [11,39]. The diminution of protein content in plants serves as a bioindicator of pollution in the environment [45]. In our study, the IBU treatments decreased the protein content of *V. unguiculata* (Figure 3G). This result may be attributed to an enhanced rate of protein denaturation caused by ROS activities and increased sodium content; the finding is consistent with the research of Hasan, et al. [46] and Munns [35]. The correlations between parameters are shown in the principal component analysis (PCA) biplot in Figure 4. The variables in group I and group II are significantly positively correlated to their own group and significantly negatively correlated with the opposite group.

IBU is a microcontaminant with relatively high solubility and intermediate hydrophobicity [47], these properties facilitate its mobility in wastewaters and its potential uptake by plants and translocation into the xylem and phloem [47,48]. In the present study, we found that IBU uptake by *V. unguiculata* progressively enhanced with increasing concentrations of IBU (Figure 3H). Low levels of IBU were bioconcentrated in the roots (6–7%) and shoots (0.2–0.9%) of *V. unguiculata* compared to the IBU concentration in the media. The amount of bioaccumulated IBU varied between 7% and 8% (Table 1). IBU was transported upward from the medium to the seeds and further translocated through roots to shoots with a translocation factor between 3% and 16%. Plants can take up, translocate, and degrade IBU by transforming IBU to eight phase-I and 38 Phase-II metabolites in the intercellular compartments, storing those compounds in the vacuoles or cell walls [26]. The uptake and metabolization of IBU to IBU-hydroxylated derivatives was also observed in *V. unguiculata* [26], *Lemna gibba* [49], and *Phragmites australis* [50].

## 3. Materials and Methods

### 3.1. Plant Growth Experiment

*V. unguiculata* L. seeds were surface sterilized using a diluted hypochlorite solution (1:10 dilution of Clorox-R and water V/V) for 20 min, rinsed repeatedly with distilled water, and then air-dried for one hour. The seeds were sown in a tray filled with perlite. After 10 days, the seedlings were transplanted into pots (Polyvinyl chloride pots; 15 cm diameter, 15 cm depth) filled with perlite. Plants were sown at a density of one seedling per pot with three replicates per treatment, including the control (acid-washed sand). The seedlings were irrigated thrice per week with a graduated treatment regimen (0, 400, 800, 1200, 1600, 2000 ppm IBU) throughout the experiment. Additional water with 300 mL of full-strength Hoagland nutrient solution was provided on alternate days to ensure adequate nutrition. In the growth chamber, photosynthetically active radiation (67–170 µmoL m^−2^ s^−1^) was provided on a 14:10 light/dark cycle; temperature (day 26 °C, night 20 °C) and relative humidity (±45%) was maintained throughout the experiment. The plants were sampled at 50 days after transplantation to assess the selected growth, morphological, and physicochemical parameters.

Length, fresh and dry weight of shoots and roots were measured at the sampling time [51]. The total leaf area (LA) of each plant was determined using an automatic leaf area meter (CI-202 area meter, CID, Inc., Camas, WA, USA).

### 3.2. Physicochemical Analysis

#### 3.2.1. Chlorophyll and Carotenoid

The chlorophyll (Chl) and carotenoid (Car) contents of leaves were measured and calculated following the method described by Lichtenthaler and Welburn [52], Porra [53]. A mortar and pestle were used to grind 0.1 g of fresh leaf with 80% acetone to extract the pigments. The extracts were passed through filter paper and the absorbance of each sample was measured at 663, 646, and 470 nm using an ultraviolet-visible spectrophotometer (UV-1800, Shimadzu, Columbia, MD, USA). Chlorophyll a, b, carotenoid, and total chlorophyll contents were calculated using the following equations.

(1) Chla=12.21A663−2.81A646

(2) Chlb=20.13A646−5.03A663

(3) $$Car=\frac{1000A470-3.27Chla-104Chlb}{229}$$

(4) Total Chl=17.76A646+7.34A663

#### 3.2.2. Ca, K, Mg, Mn, and Na Content

The experiment samples were prepared according to standard digestion procedures for plant materials [54]. In brief, 200 mg of finely ground seedling samples was added to a 100 mL TECAM digestion flask with 0.5 mL of sulfuric acid, 1 mL of perchloric acid, and 5 mL of nitric acid. The flasks were heated at 110 °C, gradually increasing to a final temperature of 330 °C. After cooling, the solution was transferred to a 50 mL calibrated flask and filled to volume with double distilled water. Standard elements were prepared, and Ca, K, Mg, Mn, and Na content were measured using a Perkin Elmer AAS-300 atomic absorption spectrophotometer according to Katz and Jennis [55].

#### 3.2.3. Hydrogen Peroxide Content

Hydrogen peroxide levels were determined following the method of Velikova et al. [56]. Plant tissues (100 mg) were homogenized in an ice bath with 5 mL 0.1% (*w/v*) trichloroacetic acid (TCA). The homogenates were centrifuged at 12,000 rpm for 15 min. Next, 0.5 mL of the supernatant was added to 0.5 mL of 10 mM potassium phosphate buffer (pH 7.0) and 1 mL of 1 M KI. The absorbance was measured at 390 nm. The H_2_O_2_ content is presented on a standard curve and expressed as nmol g^−1^ FW.

#### 3.2.4. Malondialdehyde (MDA) Assay

The level of lipid peroxidation was determined by measuring 2-thiobarbituric acid-reactive metabolites, mainly MDA, following the modified method by Heath and Packer [57]. Frozen 100 mg samples were homogenized in a pre-chilled mortar and pestle with two volumes of ice-cold 0.1% (*w/v*) trichloroacetic acid (TCA) and centrifuged for 15 min at 15,000 rpm. The 3 mL assay mixture containing 1 mL of supernatant and 2 mL of 0.5% (*w/v*) thiobarbituric acid in 20% (*w/v*) TCA was heated to 95 °C for 30 min and then rapidly cooled in an ice bath. After centrifugation (10,000 rpm for 10 min at 4 °C), the supernatant absorbance (λ = 532 nm) was read and the values corresponding to non-specific absorption (λ = 600 nm) were subtracted. MDA concentration was calculated using the following equation with an extinction coefficient of 155 mM^−1^ cm^−1^.

(5) $$MDA\;content\;\left(mM\right)=\frac{\left(A_{532}-A_{600}\right)}{155}\times vol\;of\;reaction\;mixture$$

#### 3.2.5. Enzyme Activity and Soluble Protein

Enzymes were extracted using the Beauchamp and Fridovich [58] method. A 0.1 g sample of plant material was ground with 100% *w/w* insoluble polyvinyl-polypyrrolidone in 1 mL of extraction buffer (KH_2_PO_4_/K_2_HPO_4_ 66 mM; ethylenediaminetetraacetic acid (EDTA), 1 mM; pH 7). To extract ascorbate and glutathione, sample material (0.1 g) was ground in 1 mL of 6% *w/v* metaphosphoric acid containing 1 mM EDTA. The homogenate was centrifuged (10,000 g) for 3 min at 4 °C (3K-18 centrifuge, Sigma, Osterode am Harz, Germany). Residual leaf extracts were decanted into fresh Eppendorf tubes for subsequent analysis. Superoxide dismutase (SOD) activity was assayed by measuring the ability of the enzyme extract to inhibit the photochemical reduction of nitroblue tetrazolium (NBT) to purple formazan, measured spectrophotometrically at 560 nm. Enzyme extract (0.1 mL) was added to the reaction mixture of 2.9 mL 50 mM K phosphate buffer (pH 7.8), containing 10 mM methionine, 168 µM NBT, 0.025% Triton X-100, and 1.17 µM riboflavin, for a total volume of 3 mL. The assay was placed in a test tube under yellow light for 15 min. The amount of methionine-mediated formazan formed (At) was compared with the amount of formazan formed in the absence of the enzyme (Ac). One unit of SOD is defined as the quantity that causes 50% inhibition of formazan formation. The activity was expressed as Unit activity (U mL^−1^) [58].

(6) $$SOD\;Activity\;\left(U\;mL^{-1}\right)=\frac{Ac-At}{Ac\times 0.5}$$

(7) $$\mathrm{Specific}\;\mathrm{activity}\left(\mathrm{UA}/g\;\mathrm{Protein}\right)=\frac{\mathrm{Unit}\;\mathrm{activity}\left(U/\min/g\;\mathrm{Fresh}\;\mathrm{Weight}\right)}{\mathrm{Protein}\;\mathrm{content}\left(\mathrm{mg}/g\;\mathrm{Fresh}\;\mathrm{Weight}\right)}$$

The estimation of catalase activity was performed according to the method of Aebi [59]. The assay contained 66 mM KH_2_PO_4_/K_2_HPO_4_ (pH 7), 35 mM H_2_O_2_, and 20 µL extract. Catalase activity (CAT) was determined from the decline in absorbance at 240 nm and 25 °C, following H_2_O_2_ breakdown. The quantity of enzyme necessary to release half the peroxide oxygen from hydrogen peroxide represents one unit of catalase activity. An extinction coefficient of 39.4 mM^−1^ cm^−1^ for H_2_O_2_ at 240 nm was used to calculate the activity of catalase using the following formulas.

(8) $$\mathrm{Unit}\;\mathrm{activity}\left(\frac{\frac{U}{\min}}{g}\mathrm{Fresh}\;\mathrm{Weight}\right)=\frac{\mathrm{Change}\;\mathrm{in}\frac{\mathrm{absorbance}}{\min\mathrm{Totalvolume}}\left(\mathrm{mL}\right)}{\varepsilon \times \mathrm{Volume}\;\mathrm{of}\;\mathrm{sample}\;\mathrm{used}\left(\mathrm{mL}\right)\times \mathrm{Fresh}\;\mathrm{Weight}\;\mathrm{of}\;\mathrm{sample}},$$

where ε represents extinction coefficient = 39.4 mM^−1^ cm^−1^.

(9) $$\mathrm{Specific}\;\mathrm{activity}\left(\mathrm{UA}/g\;\mathrm{Protein}\right)=\frac{\mathrm{Unit}\;\mathrm{activity}\left(U/\min/g\;\mathrm{Fresh}\;\mathrm{Weight}\right)}{\mathrm{Protein}\;\mathrm{content}\left(\mathrm{mg}/g\;\mathrm{Fresh}\;\mathrm{Weight}\right)}$$

Peroxidase was assayed following the method of Tiedemann [60]. The reagent mixture contained 0.05% guaiacol (440 µL^−1^) and 10 mM H_2_O_2_ dissolved in 25 mL sodium phosphate buffer (pH 7). Crude enzyme extracts (20 µL) and 200 µL reagent mixture were transferred into 96-well microtiter plates, then incubated for 10 min at room temperature in the dark. The absorbance of the brown guaiacol reaction was immediately measured at 450 nm, as described above. As the incubation time, sample volumes, and leaf disk areas were equal in all samples, the peroxidase (POX) activities of different samples were expressed as absorbance units (absorbances × 1000) and compared directly. POX activities were expressed as absorbance g^−1^ FW.

Glutathione reductase (GR, EC1.6.4.2) was assayed by measuring the change in absorbance at 340 nm following the oxidation of reduced Nicotinamide adenine dinucleotide phosphate (NADPH) [61]. The reaction mixture contained 100 mM potassium phosphate buffer (pH 7.0), 1.0 mM EDTA, 50 μM NADPH, 100 μM oxidized glutathione, and 100 μL enzyme in a final volume of 3 mL. GR activity was expressed as U mg^−1^ protein. The decrease in absorbance per minute was observed at intervals of 3 s at 340 nm and 25 °C. One unit of GR enzyme activity is defined as the amount of enzyme required to oxidize 1.0 μM of NADPH min^−1^ g^−1^ FW. The unit activity and specific activity were calculated using the following formulas.

(10) $$\mathrm{Unit}\;\mathrm{activity}\left(\frac{\frac{U}{\min}}{g}\mathrm{Fresh}\;\mathrm{Weight}\right)=\frac{\mathrm{Change}\;\mathrm{in}\frac{\mathrm{absorbance}}{\min\mathrm{Totalvolume}}\left(\mathrm{mL}\right)}{\varepsilon \times \mathrm{Volume}\;\mathrm{of}\;\mathrm{sample}\;\mathrm{used}\left(\mathrm{mL}\right)\times \mathrm{Fresh}\;\mathrm{Weight}\;\mathrm{of}\;\mathrm{sample}},$$

where ε represents extinction coefficient = 6.22 mM^−1^ cm^−1.^

(11) $$\mathrm{Specific}\;\mathrm{activity}\left(\mathrm{UA}/g\;\mathrm{Protein}\right)=\frac{\mathrm{Unit}\;\mathrm{activity}\left(U/\min/g\;\mathrm{Fresh}\;\mathrm{Weight}\right)}{\mathrm{Protein}\;\mathrm{content}\left(\mathrm{mg}/g\;\mathrm{Fresh}\;\mathrm{Weight}\right)}$$

Ascorbate peroxidase (APX) activity was determined from the decrease in absorbance measured at 290 nm, following the consumption of ascorbic acid (AA) [62]. APX activity was calculated using an extinction coefficient of 2.8 mM^−1^ cm^−1^ for AA at 290 nm.

Soluble proteins were assayed according to the method of Bradford [63] using bovine serum albumin as a standard and related to plant dry weight. Leaf samples (0.1 g) were ground using 3 mL of 50 mM potassium phosphate buffer with pH 7.0 phosphate buffer in an ice bath. The homogenates were centrifuged for 20 min at 13,000 rpm and 4 °C. Then, 0.1 mL of the supernatants was treated with 2 mL of Bradford reagent (50 mg of Coomassie Brilliant Blue G-250 in 50 mL of methanol, 100 mL 85% (*w/v*) phosphoric acid (H_3_PO_4_), and 850 mL of distilled water) and incubated for 5 min before absorbance was measured at 595 nm.

#### 3.2.6. Ibuprofen Analysis

The extraction, separation, concentration, and validation of IBU from experiment samples were analyzed according to Picó et al. [26] and Andreotti et al. [64] using ultra-high performance liquid chromatography (Agilent 1260 Infinity, Waldbronn, Germany) and an AB SCIEX TripleTOF™ 5600 mass spectrometer (AB SCIEX, Foster City, CA, USA). Data acquisition processing and instrument control were performed using Analyst, Peak View 1.0, and MultiQuant 2.0. software [26].

The bioconcentration factor (BCF), bioaccumulation factor (BAF), and translocation factor (TF) of IBU in *V. unguiculata* plants were calculated according to Wang [65]:

(12) $$BCF\;\left(L/Kg\right)=\frac{IBU\;concentration\;in\;media\;\left(mg/L\right)}{IBU\;concentration\;in\;plant\;tissue\;\left(mg/Kg\right)},$$

(13) $$BCF\;\left(L/Kg\right)=\frac{IBU\;concentration\;in\;media\;\left(mg/L\right)}{IBU\;concentration\;in\;whole\;plant\;tissue\;\left(mg/Kg\right)},$$

(14) $$TF=\frac{IBU\;concentration\;in\;shoot\;\left(mg/Kg\right)}{IBU\;concentration\;in\;root\;\left(mg/Kg\right)}.$$

### 3.3. Statistical Analysis

All experimental data were analyzed by one-way analysis of variance with the Bonferroni correction for multiple comparisons (*p* < 0.05) using statistical software package STATISTIX 10 (Analytical Sofware, Tallahassee, FL, USA). Regression was analyzed using PASW Statistics 18 (IBM, New York, NY, USA). Principal Component Analysis (PCA) was performed using XLstat (Addinsoft, New York, NY, USA). Graphics were produced using Microsoft Excel.

## 4. Conclusions

*Vigna unguiculata* plants develop several morphological and physicochemical adaptations to cope with ibuprofen stress. The ecotoxicological effects of IBU on plant growth consist of decreased plant length, biomass, LA, chlorophyll and carotenoid biosynthesis, impaired mineral balance, and lowered total protein. IBU uptake increased the levels of ROS such as H_2_O_2_, which were alleviated through increased synthesis of antioxidant enzymes such as SOD, CAT, POX, GR, and APX. According to the IC50 values for biomass (IBU 1253 ppm) and plant length (IBU 1955 ppm) observed in this study, we concluded that the current range of IBU environmental pollution (0.5–6 ppb) would have minimal effect on *V. unguiculata*. For future research possibilities, it would be interesting to study IBU effects on the anatomy of the plant, the location of metabolite degradation, and immobilization in cell components, the promotion of growth by low concentrations of IBU, and the genetic mechanism that controls the transformation of IBU metabolites in the plant.

## Figures and Tables

**Table 1:** The bioconcentration factor (BCF), bioaccumulation factor (BAF), and translocation factor (TF) of ibuprofen (IBU) in *V. unguiculata* plants on day 50.

	Bioconcentration Factor (BCF)		Bioaccumulation Factor (BAF)	Translocation Factor (TF)
IBU Treatments (ppm)	Roots	Shoots	Whole Plants	From Roots to Shoots
400	0.078	0.002	0.08	0.029
800	0.071	0.005	0.076	0.072
1200	0.061	0.008	0.07	0.132
1600	0.059	0.009	0.068	0.155
2000	0.06	0.01	0.07	0.162
